# Unusual Presentation of Galactosemia in a Child: Musculoskeletal Manifestations

**DOI:** 10.31138/mjr.30.2.123

**Published:** 2019-06-29

**Authors:** Faiq I. Gorial, Maab Jasim Mohammed

**Affiliations:** 1 Rheumatology Unit, Department of Medicine, College of Medicine, University of Baghdad, Baghdad, Iraq; 2 Rheumatology Unit, Baghdad Teaching Hospital, Baghdad, Iraq

**Keywords:** Galactosemia, musculoskeletal manifestations, bone mineral density

## Abstract

Galactosemia is an autosomal recessive inherited disease of galactose metabolism. In this report, a galactosemia case with unusual presentation has been presented. We reported a child boy with galactosemia presented with arthralgia, hands deformity and decreased bone mineral density.

## INTRODUCTION

Galactosemia is an autosomal recessive inherited disease of the galactose metabolism due to galactose-1-phosphate uridyl transferase deficiency.^1^ Classic galactosemia is characterized by vomiting, diarrhoea, anaemia, jaundice, failure to thrive, hypoglycaemia, hepatosplenomegaly, hepatocellular insufficiency, renal tubular dysfunction, cataracts, hypotonia and sepsis.^2,3^ Long-term complications such as a decreased bone mineral density (BMD) might be the result of either dietary deficiencies secondary to the galactose-restricted diet or unknown intrinsic factors.^4^

## CASE REPORT

A six-year-old boy was pre-diagnosed with galactosemia during infancy when he developed vomiting, poor feeding, jaundice, liver dysfunction and failure to thrive. Serum total bilirubin (8.7 mg/dl, reference range 0.3–1 mg/dl) was elevated. Plasma aspartate transaminase (89 U/L, reference range <20 U/L), alanine transaminase (94 U/L, reference range <20 U/L), and alkaline phosphatase (400 U/L, reference range 30–85 U/L) were significantly elevated. Liver biopsy was performed and revealed degeneration of hepatocyte with giant cell transformation and moderate cholestasis of both hepatocellular and canalicular with bile thrombi associated with minimal necro-inflammatory changes of hepatocyte. Galactose-1-phosphate uridyl transferase (GALT) (1.7 U/g, reference range >3 U/g) was deficient. During infancy, he was managed by removing all galactose from the diet as soon as the diagnosis was suspected, immediately after starting the diagnostic investigations and without awaiting results, in order to prevent further life-threatening complications.

There was family history of galactosemia of third-degree relative (cousin).

**Figure 1.**
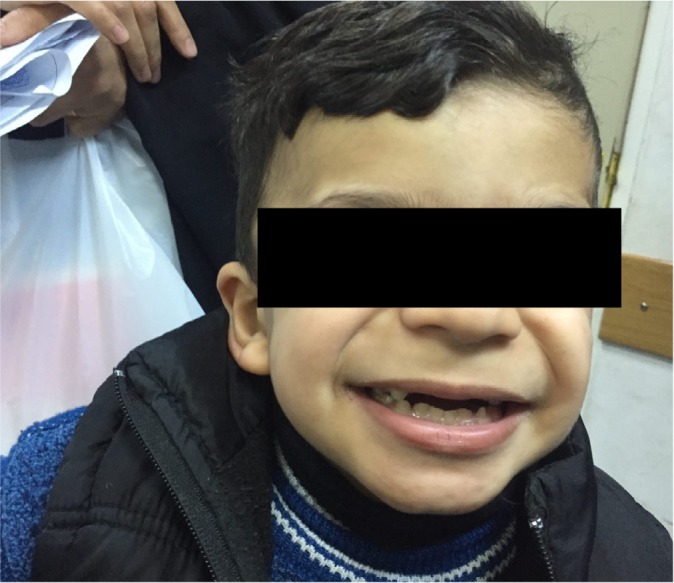
Poor dentition.

**Figure 2.**
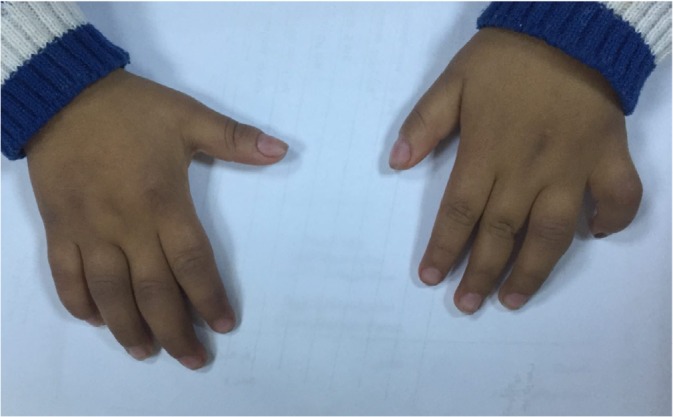
Hands manifestations: dark skin pigmentation over the small joints of the hands, widening of the fingers most obvious in the ring and little fingers of both hands.

The young patient presented for examination because of joints pain and deformities. On examination, he was of short stature with poor dentition (*Figure 1*). Hands examination revealed dark skin pigmentation over the small joints of the hands, widening of the fingers most obvious in the ring and little fingers of both hands, and limitation of range of motion of the fingers (*Figure 2*). The dual x-ray absorptiometry (DXA) scan revealed osteopenia (Z score −2.2). The whole skeletal survey revealed decreased bone density with wide medulla as seen in bone marrow.

## DISCUSSION

We reported a case of galactosemia in a 6-year Iraqi boy patient. Our patient was pre-diagnosed with galactosemia based on clinical features and accessible laboratory results. Galactosemia results from an enzymatic defect of galactose metabolism and is divided into three types, including galactokinase (GALK) deficiency, galactose-1-phosphate uridyl transferase (GALT) deficiency, and galactose-6-phosphate epimerase (GALE) deficiency.^5^ The free galactose in plasma is subsequently changed into galactitol and galactonate through the alternative pathway of galactose metabolism. Accumulation of galactitol and galactonate destroy selected tissue which effects multiple organs, including liver, kidney, and brain and long-term effects including chronic brain injury, cataract, and decreased bone mineral density.^6^ Diminished mineralization of bones appears to be another abnormality associated with galactosemia, likely secondary to abnormal levels of gender steroids in female patients, low calcium intake, and perhaps an intrinsic defect in the normal galactosylation of the collagen matrix of bone caused by the enzyme defect.^7^

## CONCLUSION

Long-term complications occur in galactosemia. Strategies to improve bone formation should be considered to diminish morbidity in patients with this inborn error of metabolism. Follow up with DXA scan is necessary for assessment and detection of decreased bone mineral density.
